# WNT Ligand Dependencies in Pancreatic Cancer

**DOI:** 10.3389/fcell.2021.671022

**Published:** 2021-04-28

**Authors:** Kristina Y. Aguilera, David W. Dawson

**Affiliations:** ^1^Department of Pathology and Laboratory Medicine, David Geffen School of Medicine at University of California, Los Angeles, CA, United States; ^2^Jonsson Comprehensive Cancer Center, David Geffen School of Medicine at University of California, Los Angeles, CA, United States

**Keywords:** pancreatic ductal adenocarcinoma, intraductal papillary mucinous neoplasms, RNF43, PORCN, R-spondin, WNT/β-catenin signaling, WNT7B, FZD5

## Abstract

WNT signaling promotes the initiation and progression of pancreatic ductal adenocarcinoma (PDAC) through wide-ranging effects on cellular proliferation, survival, differentiation, stemness, and tumor microenvironment. Of therapeutic interest is a genetically defined subset of PDAC known to have increased WNT/β-catenin transcriptional activity, growth dependency on WNT ligand signaling, and response to pharmacologic inhibitors of the WNT pathway. Here we review mechanisms underlying WNT ligand addiction in pancreatic tumorigenesis, as well as the potential utility of therapeutic approaches that functionally antagonize WNT ligand secretion or frizzled receptor binding.

## Introduction

Pancreatic ductal adenocarcinoma (PDAC) is an aggressive and hard to treat malignancy with an overall 5-year survival of only 10%. It is currently the third leading cause of cancer mortality in the United States (Siegel et al., 2021). PDAC arises from two premalignant histologic precursors—pancreatic intraepithelial neoplasia (PanIN) or macroscopic cystic neoplasia including intraductal papillary mucinous neoplasms (IPMNs) and mucinous cystic neoplasms (MCNs). The molecular hallmark of PDAC is *KRAS* mutation, a near ubiquitous and critical oncogenic driver of tumor initiation and progression. A diverse array of additional signaling pathways and processes further contribute to pancreatic tumorigenesis (Kleeff et al., 2016; Pelosi et al., 2017). Next generation sequencing (NGS) reveals a genetically diverse landscape (averaging >60 mutations/tumor) including four high frequency drivers (*KRAS, CDKN2A, TP53*, and *SMAD4*) and many additional heterogeneous genetic alterations. NGS studies also broadly divide PDAC into (1) classical/epithelial and (2) basal/squamous/quasimesenchymal transcriptional subtypes associated with *GATA6* and *TP63* expression signatures, respectively (Jones et al., 2008; Moffitt et al., 2015; Waddell et al., 2015; Witkiewicz et al., 2015; Bailey et al., 2016; Kleeff et al., 2016; Cancer Genome Atlas Research Network [CGARN], 2017). WNT is a highly enriched molecular mechanism in PDAC based on mutational analysis and expression profiling. Altered expression and activity of upstream or downstream WNT pathway components promote cancer hallmarks linked to pancreatic cancer initiation, progression, dissemination, stemness, and therapeutic resistance (White et al., 2012; Donahue and Dawson, 2016; Makena et al., 2019; Zhong et al., 2020). Of relevance to precision oncology is a subset of PDAC with *ring finger protein 43* (*RNF43)* mutations conferring growth addiction to WNT ligands. This review briefly summarizes mechanisms of plasma membrane WNT ligand signaling in PDAC and their biological and clinical implications.

## Regulation and Function of WNT Ligand Signaling in PDAC

### Canonical WNT Ligand Signaling in PDAC

**FIGURE 1:**
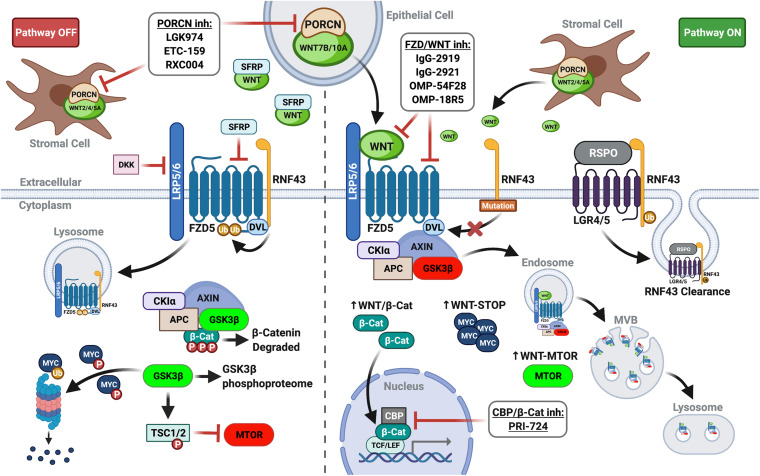
WNT plasma membrane signaling in PDAC. (Left panel) In the absence of FZD-LRP5/6 signaling (lack of WNT ligand and/or inhibition by extracellular DKKs or SFRPs), β-catenin is targeted for degradation. GSK3β activity also impinges other signaling pathways and controls ubiquitin-mediated degradation of proteins. (Right panel) Augmented by *RNF43* mutations or RSPO inhibition of RNF43, WNT ligand signaling re-localizes and inactivates the destruction complex to stabilize β-catenin, increasing its nuclear translocation and co-transcriptional activity. GSK3β sequestration in multivesicular bodies (MVB) prevents substrate phosphorylation, promoting WNT-STOP, WNT-MTOR, and further pathway crosstalk (i.e., de-repression of MAPK/ERK). Thus, upstream WNT pathway inhibitors (PORCN inhibitors, FZD receptor blockers, and FZD decoy receptors) mediate additional actions divergent from downstream WNT pathway inhibitors targeting β-catenin in the nucleus (i.e., PRI-724). Created with BioRender.com.

Canonical WNT signaling involves oligomerization of frizzled (FZD) receptor and low-density lipoprotein-receptor related protein 5/6 (LRP5/6) by WNT ligand, initiating signaling classically culminating in the stabilization and nuclear translocation of β-catenin (Nusse and Clevers, 2017). Independent of β-catenin, WNT-FZD-LRP5/6 complexes sequester glycogen synthase kinase 3-beta (GSK3β) in multivesicular bodies, preventing its phosphorylation of target substrates. Consequently, canonical WNT signaling inhibits GSK3β phosphorylation-initiated ubiquitin-mediated degradation of numerous target substrates through the WNT stabilization of proteins (WNT-STOP) process. GSK3β sequestration also impinges on other signaling pathways modulated by its phosphorylation, such as mammalian target of rapamycin signaling induced by WNT (WNT-MTOR) (Acebron and Niehrs, 2016; Figure 1).

Genetically engineered mouse models (GEMMs), isogenic PDAC cell lines, and patient-derived organoids and xenografts highlight the activity and function of canonical WNT signaling in pancreatic tumorigenesis. Canonical WNT is activated early in PanIN progression and variably across PDAC tumors and cell lines (Pasca di Magliano et al., 2007; White et al., 2012). Genetic or pharmacologic inhibition of WNT ligand signaling or β-catenin itself blocks acinar-to-ductal metaplasia, PanIN, and PDAC in mouse models, including the conditional *Kras*^LSL–G12D^ (KC) mouse model of PDAC (Zhang et al., 2013). Paradoxically, WNT hyperactivation via *Ctnnb1* stabilizing mutation also blocks PanIN formation and PDAC progression in the KC model (Heiser et al., 2008). Thus, the timing, strength, and manner of WNT activation are critical for pancreatic tumor initiation and progression in the context of oncogenic *KRAS* (MorrisIV., Wang and Hebrok, 2010). In patient samples, hallmark WNT mutations linked to constitutive pathway activation (i.e., *CTNNB1, APC*, etc.) are common primary oncogenic drivers in other non-PDAC pancreatic malignancies (i.e., acinar carcinoma and solid-pseudopapillary neoplasm) (White et al., 2012). By contrast, canonical WNT signaling in PDAC is primarily dysregulated at the plasma membrane level (White et al., 2012; Donahue and Dawson, 2016; Makena et al., 2019; Zhong et al., 2020).

The variable and complex expression patterns of FZD receptors and WNT ligands in PDAC cell lines and tissues raises important questions about functional redundancy or specificity of ligand-receptor combinations. A shared pattern of WNT1 and FZD2 expression correlates with increased total and non-phosphorylated β-catenin in PDAC tissue samples (Zeng et al., 2006). Interestingly, macrophage-derived WNT1 promotes epithelial-mesenchymal transition (EMT) in support of vascular invasion and metastasis in breast cancer (Linde et al., 2018), suggesting a WNT1-FZD2 circuit might be linked to inflammatory cell-mediated paracrine signaling in PDAC. Canonical WNT signaling mediates pancreatic stellate cell (PSC) activation and tumor-stromal crosstalk in PDAC. PSC activation correlates with downregulation of DKK1 and upregulation of WNT2 and β-catenin, while DKK1 antagonizes PSC activation and collagen synthesis (Hu et al., 2014). In organotypic models, activated PSCs secrete WNT2 to drive canonical WNT signaling in PDAC cells, while retinoic acid-induced PSC quiescence reduces WNT activation in PDAC (Froeling et al., 2011; Xu et al., 2015). Thus, paracrine WNT signaling from the tumor microenvironment can drive WNT activation in pancreatic cancer cells.

In relation to autocrine signaling, WNT7B is enriched in PDAC cell lines with high constitutive WNT/β-catenin signaling, is crucial for WNT/β-catenin transcriptional activity, and promotes *in vitro* and *in vivo* tumorigenesis (Arensman et al., 2014). WNT7B, WNT10A, and FZD5 are identified as essentiality genes in a large pooled CRISPR fitness screen of *RNF43*-mutant WNT ligand-addicted PDAC cell lines. FZD5 is therapeutically targetable specifically in *RNF43*-mutant PDAC cell lines and patient-derived xenograft (PDX) using anti-FZD5 antagonistic antibodies with limited FZD8 cross-reactivity (Steinhart et al., 2017). Further highlighting the specificity and potency of specific ligands in autocrine and paracrine signaling, Seino et al. (2018) stratify patient-derived PDAC organoids (PDOs) into three distinct subtypes: (1) growth dependent on WNT ligand provided exogenously or through co-culture with stromal cells; (2) growth sustained by autocrine WNT but sensitive to inhibitors of WNT secretion; or (3) growth independent of WNT. Further phenotypic studies and analysis of patient samples identify epithelial cell-derived (WNT7B and WNT10A) and stromal cell-derived (WNT2 or WNT2A) ligands of functional and clinical significance in PDAC. Interestingly, WNT independent PDOs consistently lack *GATA6* expression but are driven into WNT dependency via exogenous *GATA6* expression (Seino et al., 2018). GATA6 is overexpressed in precursor PanINs and promotes WNT activation and PDAC growth through transcriptional downregulation of the secreted WNT inhibitor DKK1 (Zhong et al., 2011). Thus, WNT ligand dependency is linked to GATA6 expression/function and classical/epithelial transcriptional subtype of PDAC. This transcriptional control of WNT dependency in PDAC is likely highly complex as studies in heart and lung development highlight highly interdependent expression and function of secreted WNT ligands and inhibitors, FZD receptors, and GATA transcription factors in mediating canonical and non-canonical WNT signaling (Afouda et al., 2008; Zhang et al., 2008; Meganathan et al., 2015).

### RNF43 and WNT Growth Addiction in PDAC

The ubiquitin E3 ligase RNF43 is a key WNT feedback inhibitor that downregulates canonical signaling by ubiquitinating plasma membrane FZD receptors and LRP5/6 co-receptors, resulting in their internalization and lysosomal degradation (Figure 1). Secreted R-spondin family members (RSPO1-4) inhibit this process by binding leucine-rich repeat-containing G-protein coupled receptor (LGR4/5/6) and RNF43 (Binnerts et al., 2007; Hao et al., 2012) to potentiate WNT ligand signaling. Mutational inactivation of *RNF43* confers growth dependency on autocrine WNT ligand signaling in PDAC lines and predicts response to WNT inhibitors (Jiang et al., 2013). RSPO further regulates cellular hierarchy and cancer stem cell (CSC) phenotypes in PDAC irrespective of *RNF43* mutational status. Subpopulations of PDAC cells with high intrinsic WNT activity express RSPO2, which supports EMT and stemness phenotypes enhancing tumor-initiating and metastatic potential (Ilmer et al., 2015). Thus, PDAC CSC may be further specifically targetable with antagonistic antibodies to RSPO2 or other RSPO members under clinical investigation.

Approximately 5–7% of PDAC and 15–40% of premalignant IPMNs and MCNs harbor mutations in *RNF43.* Comparatively infrequent in PanIN and PanIN-associated PDAC, *RNF43* mutations are primarily linked to the malignant progression in IPMN and MCN (Furukawa et al., 2011; Wu et al., 2011; Jiang et al., 2013; Waddell et al., 2015; Bailey et al., 2016; Cancer Genome Atlas Research Network [CGARN], 2017) and are useful ancillary markers in pancreas cyst fluid diagnostics (Springer et al., 2015). Capture-based whole exome sequencing reveals IPMNs arising as multiple heterogeneous clones with convergent evolution of *RNF43* mutations during dysplastic progression (Fischer et al., 2019). Comprehensive sequencing and functional analysis suggest most *RNF43* non-sense and frameshift mutations and missense mutations in its RING domain and N-terminal region increase WNT activity and predict *in vivo* response to upstream WNT pathway inhibition (Yu et al., 2020). Large genomic deletion of *Rnf43* by inducible CRISPR in KC mice does not lead to cystic neoplasia but does accelerate PDAC progression, implying WNT signaling also facilitates malignant progression of *Kras-*initiated PanIN (Mishra et al., 2020).

### Non-canonical WNT Signaling in PDAC

Non-canonical WNT ligand signaling in pancreatic tumorigenesis includes roles in potentiation of drug resistance and metastasis through effects of EMT and cancer stemness (Makena et al., 2019). WNT2 and other WNT ligands are upregulated in PDAC cells under anchorage-independent conditions. WNT2 suppresses anoikis and potentiates metastasis via non-canonical WNT signaling mechanisms involving fibronectin upregulation and MAP3K7 signaling. WNT2 and WNT5A are enriched in subsets of circulating tumor cells collected from PDAC patients and act as orthogonal drivers of stemness and EMT (Yu et al., 2012; Franses et al., 2020). WNT5A/WNT5B induce EMT and potentiate metastasis across multiple cancer types through FZD2 non-canonical mechanisms involving FYN and STAT3 (Gujral et al., 2014). WNT5A also signals through FZD7 to mediate gemcitabine resistance in PDAC via upregulation of *ABCG2* (Zhang et al., 2021). Upregulated in PanIN and PDAC, WNT5A mediates apoptosis resistance to chemotherapy in PDAC lines through a NFATc2 dependent-mechanism stabilizing β-catenin (Griesmann et al., 2013) highlighting complex and overlapping roles of certain WNT ligands in regulating canonical and non-canonical signaling. Indeed, non-canonical WNT signaling suppresses pancreatic tumorigenesis in certain contexts via its capacity to suppress canonical WNT at different levels. Oncogenic KRAS sequesters calmodulin, which inhibits FZD8 receptor expression to block downstream NFAT and CaMKII-mediated antagonism of β-catenin in PDAC (Wang et al., 2015). This mechanism may explain the requirement for tightly regulated patterns of KRAS and WNT signaling during PDAC initiation and progression (MorrisIV., Wang and Hebrok, 2010).

## Targeting WNT Ligand Dependency in PDAC

### Porcupine Inhibitors

Porcupine O-acyltransferase (PORCN) palmitoylates WNT ligands, a critical post-translational modification necessary for proper WNT processing, secretion, and FZD binding (Proffitt and Virshup, 2012). PORCN inhibitors (PORCNi) consistently and potently inhibit WNT/β-catenin transcription and growth of WNT-addicted cancers in both *in vitro* and *in vivo* preclinical models (Jiang et al., 2013; Liu et al., 2013; Arensman et al., 2014; Bailey et al., 2016; Hao et al., 2016; Madan et al., 2018a; Woodcock et al., 2019; Kalantary-Charvadeh et al., 2020). Justifying potential patient selection in their use, LGK974 was the first PORCNi shown to broadly block WNT/β-catenin transcriptional activity while only inhibiting growth of *RNF43*-mutant PDAC lines (Jiang et al., 2013; Figure 1). Multiple PORCNi (LGK974, ETC-159, and RXC004) have advanced to phase I or II clinical trials in advanced solid tumors alone and/or in combination with other therapies (Makena et al., 2019). Clinical trials exploring PORCNi in PDAC include NCT01351103, NCT02521844, and NCT03447470. Of note, some of these trials examine PORCNi in combination with immune checkpoint inhibitors given important roles for WNT in cancer immune escape. Targeting WNT will alter key regulators of the tumor immune cycle across tumor cells, antigen presenting cells, and different T cell subsets and has the potential to overcome primary, adaptive, and acquired resistance mechanisms to cancer immunotherapy (Wang et al., 2018).

Porcupine O-acyltransferase inhibitors have been leveraged as tool compounds for in-depth functional studies of WNT ligand signaling in PDAC. In a comprehensive study of *in vitro* and *in vivo* transcriptional dynamics, ETC-159 modulated >20% of expressed genes in *RNF43*-mutant PDAC lines. Altered genes were enriched for targets in cell cycle, nucleic acid metabolism, and ribosomal biogenesis. This transcriptional remodeling involved GSK3β-dependent regulation of β-catenin and MYC, the latter partially mediated via WNT-STOP mechanism independent of β-catenin (Madan et al., 2018a). This provocative finding suggests one critical mechanism of WNT addiction in PDAC may be the stabilization of proteins other than β-catenin (Figure 1). An *in vivo* CRISPR loss-of-function screen with ETC-159 identifies PI3K/mTOR pathway as a synthetic vulnerability. ETC-159 combined with pan-PI3K/mTOR inhibitor GDC-0941 more potently suppresses *in vitro* and *in vivo* PDAC tumorigenesis by enhancing cell cycle arrest, cellular senescence, and reduced glucose metabolic flux (Zhong et al., 2019). PORCNi leads to augmented MAPK and JNK activity in PDAC lines. ETC-159 combined with MEK inhibitor Trametinib synergistically inhibits cell cycle progression and *in vivo* tumor growth of *RNF43*-mutant PDAC, leading study authors to propose that WNT may temper excessive and potentially deleterious MAPK/ERK signaling in *RNF43*-mutant PDAC (Zheng et al., 2021).

### Anti-FZD Antibodies and Fusion Protein Decoys

The monoclonal antibody OMP-18R5 (Vantictumab) antagonizes WNT signaling by binding multiple FZD receptors (FZDs 1, 2, 5, 7, and 8). OMP-18R5 is effective against PDAC in transgenic and xenograft models alone or synergistically with cytotoxic therapies, including gemcitabine or nab-paclitaxel (Gurney et al., 2012; Zhang et al., 2013; Fischer et al., 2017; Steinhart et al., 2017). Its safety has been evaluated in multiple cancer types, including PDAC (Smith et al., 2013). A phase Ib trial evaluating OMP-18R5 with nab-paclitaxel and gemcitabine in untreated metastatic pancreatic adenocarcinoma (NCT02005315) observed partial disease response in 41.9% and stable disease in 35.5% of patients, a potentially modest improvement over chemotherapy alone. However, definitive conclusions about efficacy were limited by study design and early study termination prior to reaching maximal tolerated dose (Davis et al., 2020).

OMP-54F28 (Ipafricept) (Figure 1) is a first-in-class recombinant protein fusing an extracellular portion of human FZD8 receptor to human IgG1 Fc fragment. It acts as a decoy receptor for WNT ligands (Le et al., 2015; Jimeno et al., 2017; Moore et al., 2019). OMP-54F28 alone is more effective than gemcitabine and improves efficacy with paclitaxel in preclinical PDX models (Fischer et al., 2017). A phase Ib trial evaluating OMP-54F28 with nab-paclitaxel and gemcitabine finds an overall response rate (ORR) of 35% and clinical benefit rate of 81%. Although a potentially modest improvement over chemotherapy alone, definitive conclusions regarding the efficacy of OMP-54F28 were limited by study design and early termination due to concerns surrounding safety and commercial viability (Dotan et al., 2019).

### Therapeutic Caveats

On-target effects linked to disruption of WNT and its role in normal homeostasis are concerns for PORCN and FZD inhibitors. Disrupted bone homeostasis is the most serious clinical toxicity observed to date with fragility fractures observed as a significant adverse event with OMP-18R5 (Smith et al., 2013; Tai et al., 2015). OMP-18R5 and OMP-54F28 clinical trials in PDAC were terminated due to concerns surrounding bone complications and commercial viability given an overall lack of therapeutic index across multiple studies (Moore et al., 2019; Davis et al., 2020). Although generally tolerated, PORCNi reduces bone mineral density, strength, and volume in mice by disrupting the balance of adipocytes and osteoblasts arising from mesenchymal stem cells (Funck-Brentano et al., 2018; Madan et al., 2018b). The DNA methylation inhibitor 5-Aza-dC mediates anti-adipogenic and pro-osteoblastogenic phenotypes that are reversible with PORCNi. These phenotypes appear linked to disruption of WNT10A and its regulation of mesenchymal stem cell fate (Chen et al., 2016). As a mitigating strategy, co-administration of the anti-resorptive bisphosphonate alendronate with ETC-159 reverses bone mass loss by rebalancing the activity of osteoclasts and preventing accumulation of bone marrow adipocytes (Funck-Brentano et al., 2018; Madan et al., 2018b). The addition of bone monitoring and bone protective agents with PORCN and FZD inhibitors have been employed in clinical trials although the relative benefit of these mitigating approaches remain uncertain. As an aside, some on-target effects may be clinically desirable. For example, PORCNi ameliorates chemotherapy-induced neuropathic pain via antagonism of canonical WNT ligand signaling in nerve and dorsal root ganglion in rodent models (Resham and Sharma, 2019; Kim et al., 2020) and could benefit patients on chemotherapies with dose-limiting neuropathies.

## Discussion

WNT ligand signaling plays key roles in PDAC initiation, progression, dissemination, and therapeutic resistance. Despite exciting results in preclinical models and identification of a subset of PDAC tumors addicted to WNT ligand, the safety and efficacy of upstream WNT inhibitors is uncertain. Adoption of mitigation strategies for dose-limiting side effects and biomarker-driven patient selection could enhance therapeutic index. Additionally, co-administration of highly specific WNT agonists such as next-generation surrogate WNTs that heterodimerize specific FZD isoform-LRP6 combinations (Miao et al., 2020) could facilitate on-target rescue of WNT signaling linked to toxicity while broadly inhibiting WNT systemically with PORCNi or FZD antagonists. Novel approaches such as drug conjugates or functionalized nanoparticles can also be envisioned for targeted delivery of WNT inhibitors specifically to the PDAC tumor microenvironment. Opportunities also exist to leverage known or novel drug combinations targeting tumor cell-specific vulnerabilities elicited by WNT inhibitors, including MEK or MTOR inhibition in combination with PORCNi. Finally, clinically effective and safe WNT inhibitors may ultimately hinge on the development of agents that selectively target PDAC-specific WNT-FZD circuits identified through functional approaches and spatiotemporal analyses of PDAC.

## Author Contributions

KYA and DWD conceptualized and wrote the manuscript. Both authors contributed to the article and approved the submitted version.

## Conflict of Interest

The authors declare that the research was conducted in the absence of any commercial or financial relationships that could be construed as a potential conflict of interest.
